# The Regeneration of the Pulp

**Published:** 1903-02

**Authors:** Arthur R. Dray

**Affiliations:** Philadelphia


### International Dental Journal.

Vol. XXIV. February, 1903. No. 2.

### Original Communications.[1]

### THE REGENERATION OF THE PULP.[2]

BY DR. ARTHUR R. DRAY, PHILADELPHIA.

I feel that I am taking much upon myself by appearing before you to-night, surrounded as I am by so many who could have presented a more acceptable and profitable paper. It is not in my mind to read or say anything for your edification. On the contrary, I have long since wished to have some light thrown upon a subject that has vexed me not a little, and I feel that I could not do better than bring my troubles to you.

The subject of my paper is "The Regeneration of the Pulp." While it really is my subject, it does not cover the entire ground that I am going to touch upon. The old bug-bear of dentists, old and young, is, I think, that knotty question, "pulp-canal filling," and it is with this topic in view that I wish to consider "The Regeneration of the Pulp."

The many methods of pulp-canal filling have been so frequently and ably demonstrated by so many eminent men that to-day "pulp-

[1] The editor and publishers are not responsible for the views of authors of papers published in this department, nor for any claim to novelty, or otherwise, that may be made by them. No papers will be received for this department that have appeared in any other journal published in the country.

[2] Read before the Academy of Stomatology, June 24, 1902.

canal filling" is considered a matter of fact, and a more or less easy operation; but at the same time the truth remains that the trials are many when a successul root-canal filling is the goal sought for. Access to canal; location of canal; size, position, and direction of canal; entire removal of pulp-canal contents; tortuosity of canal, etc., and when the canal is free from all pulp-tissue and débris the battle is only begun, for the filling of the canal is, after all, the most tedious part of the operation. We have heard of the many forms of root-canal filling-materials, from the simple cotton to the gold-foil. They all have their advocates. Notwithstanding the many methods for canal preparation and filling, and the records of success, the past knows of many unrecorded as well as of many recorded disastrous failures. In the hands of the very best many an obdurate tooth has refused to respond favorably, many a patient has been lost, many a restless night caused, and many a regret on both sides expressed.

I am not here to point out the redeeming points of this or that method or material, but to suggest one more method,—namely, to allow the regenerated pulp to be the root-filling. I do not lay claim to originality in this method, but at the same time I must say that I have not succeeded in finding any literature on the subject, nor have I succeeded in finding any who have thought of the same from a practical point of view. While adding to the list of methods which is already legion is in a sense making matters still more complicated, I lay claim to simplicity inasmuch as permitting the regenerated pulp to fill the canal is, so far as human efforts are concerned, simplicity itself.

The regeneration of the different tissues of the animal economy is as old as man, and nature when injured puts forth a silent effort to repair defects. Surgery from its earliest conception has to a more or less degree made use of this fact. This holds as good in dentistry as it does in surgery.

A little over a year ago I was assisting at an operation that Professor Laplace, of the Medico-Chirurgical College, was performing for tic douloureux, and which consisted of the removal for the second time of the inferior dental nerve. A few days previous to that time I had had charge of a very aggravating case of apical abscess due to defective root-filling. I had taken all the precautions and care that I knew of, and yet met with ignominious defeat, and was nigh catching at a straw, as it were, for help. At the next appointment with my patient, having previously relieved the abscessed condition by the removal of the root-filling, which was of the ordinary gutta-percha variety, and having dressed the tooth once or twice, I isolated the same, which was by this time in a normal condition and free from all soreness. Using the rubber dam, I very carefully sterilized all parts with creosote and a mild bichloride solution; then, after drying, I moistened the pulp-cavity and canal with sterilized glycerin. I then capped the canal-chamber as though I were capping a live pulp, hermetically sealing all with Harvard cement. My patient has never had any trouble with the tooth since, and I am satisfied that that canal has regenerated pulp in its interior, as the tooth responds to heat and cold and in all respects acts like the so-called live tooth. Since then I have tried numerous experiments on pulps with gratifying results.

I will mention one instance that gave me great satisfaction. I had a very sensitive molar that I wished to cover with a crown. The tooth had a live pulp, and my patient would not permit me to cut it to shape, so I resorted to extirpation of the pulp, which was perfectly healthy, by the pressure method, using vapocaine. I here also capped the pulp-chamber and canal as mentioned in the previous case. My patient then permitted me to cut away all the tooth-structure that I desired to remove, and I crowned it. So far all is well, and the crowned tooth has given no trouble.

The last case I will mention was the most successful, or rather the most convincing of all. I was preparing a cavity in a superior lateral incisor for a gold filling, when I accidentally broke through into the pulp-chamber. I immediately removed the pulp and again capped over the empty chamber and root-canal and put in the gold filling at the same sitting. A month afterwards I was filling the same tooth on the distal surface, when I again accidentally drove an instrument into the pulp-chamber. The pain experienced by my patient was very severe and hemorrhage was profuse. I removed pulp number two from this one and the same tooth, and not wishing to run any further risks with my patient, I filled the root-canal and cavity at the same sitting. I have given you three successful cases; I have had two complete failures, but I feel sure that the fault was in the manipulation rather than in the method. In one of the faulty cases I had suppuration; in another severe pain was experienced very much like that of the ordinary hyperæmia of the pulp.

As I said before, I am not here to do any teaching, and I do not make any positive assertions, but I do feel that the topic that I have presented this evening is one worthy of consideration. I am still doing a little work in this line, and trust to continue the study with the hope of being able to report better success at a later date. I am exceedingly sorry to say that my histological knowledge of the regenerated pulp is slight, being based chiefly upon the rough examination of the regenerated pulp of the second case I have cited. I found the pulp highly sensitive and exceedingly vascular, and consisting largely of a body tissue resembling the embryonic connective variety, granulated in places, with the blood-vessels and nervous elements well demonstrated. One point puzzled me, and that was the outer surface or coat of the organ. I could not make out what the covering was; it seemed well defined and to all appearance was epithelial in variety, and yet I do not understand how that could be.

Dr. Black claims that hypertrophied pulp is covered with epithelium, but I cannot satisfy myself here either, not being able to accept his theory that this epiblastic tissue comes from the epithelium on the gums by grafting. The blood-clot, should there be any, which is very possible, plays no part in the formation of the new tissue, but is supplanted by cellular infiltration, which forms fibrilar connective tissue by proliferation of endothelium of vessels. As to whether the neuroplasm of the main nerve-trunk is responsible for the formation of new nervous tissue, or what the functional activity of the regenerated organ is, I cannot say anything as yet. I do not see how it can be credited with the ability to form secondary dentine, and yet we cannot say that the odontoblastic layer of cells does not reappear with the rest of the regenerated organ. The second pulp can surely calcify, should nature care to bring about such action.

The advantages of a regenerated pulp lie in our being spared the time and trouble of filling the root or roots and in keeping the tooth alive internally as well as externally.

Have not dentists been too fearful about exposing and treating pulps? The timidity has been brought about by repeated failures and sorry consequences, but can we not trace it to the same cause to which the surgeon attributed the one-time hospital gangrene,—namely, the lack of antiseptic principles?

Pulp and pulp-canals should be treated strictly antiseptically as to instruments, hands, rubber dam, medicaments, and all materials; in short, as does the surgeon so should do the dentist.

